# Effects of massage therapy for patients with thoracic facet joint disorders: A protocol for systematic review and meta-analysis

**DOI:** 10.1097/MD.0000000000023480

**Published:** 2020-12-04

**Authors:** Ke-Lin Zhou, Shuo Dong, Wei Ji, Jing-Yi Yang, Mei-Ling Ren, Bao-Lai Mi, Sheng Guo, Ming-Heng Cai

**Affiliations:** a Dongfang Hospital of Beijing University of Chinese Medicine; b School of Traditional Chinese Medicine, Beijing University of Chinese Medicine, Beijing, China.

**Keywords:** complementary and alternative medicine, massage therapy, protocol, thoracic facet joint disorder

## Abstract

**Background:**

Thoracic facet joint disorder is a common thoracic disorder in clinic, inducing pain and discomfort at the dislocated thoracic vertebrae, radiating to pain of the neck and back. The incidence of thoracic facet joint disorder is higher than the facet disorder of the cervical and lumbar vertebrae. Therefore, an ideal strategy to relieve thoracic facet joint disorder is urgently needed. In recent years, massage therapy has been increasingly accepted by thoracic facet joint disorder patients due to its lower costs, fewer unwanted side effects and safety for clinical use. In this systematic review, we aim to evaluate the effectiveness and safety of massage therapy for patients with thoracic facet joint disorder.

**Methods:**

We will search the following electronic databases for randomized controlled trials to evaluate the effectiveness of massage therapy in treating thoracic facet joint disorder: Wanfang and PubMed Database, CNKI, CENTRAL, CINAHL and EMBASE. Each database will be searched from inception to October 2020. The entire process will include study selection, data extraction, risk of bias assessment and meta-analyses.

**Results:**

This proposed study will evaluate the effectiveness of massage therapy for patients with thoracic facet joint disorder.

**Conclusions:**

This proposed systematic review will evaluate the existing evidence on the effectiveness and safety of massage therapy for patients with thoracic facet joint disorder.

**Dissemination and ethics:**

The results of this review will be disseminated through peer-reviewed publication. Because all of the data used in this systematic review and meta-analysis has been published, this review does not require ethical approval. Furthermore, all data will be analyzed anonymously during the review process.

**OSF Registration number:**

DOI 10.17605/OSF.IO/XMEJD

## 1 Introduction

Thoracic facet joint disorder syndrome is one of the common spinal disorders, also named as facet joint disorder of thoracic vertebrae, disorder of posterior joint of thoracic vertebrae or dislocation of posterior joint of thoracic vertebrae. It includes a series of symptoms with nerves, soft tissues and internal organ involved, such as pain of chest and back, stuffiness in chest, palpitations, cough and gastrointestinal dysfunction.^[1,2]^ Thoracic facet joint disorder generally occurs at the 3rd to 6th thoracic vertebrae caused by the anatomical positional change of thoracic facet joint and is one of the common factors of chest and back pain.^[3,4]^ When external force acts on thoracic vertebrae, the intervertebral facet joints (posterior thoracic joints and costovertebral joints) will slightly subluxate and the synoviums of joints will be stuck into the subluxated articular cavity under the action of negative pressure, leading to pain and dysfunction.^[5,6]^

However, due to the lack of attention to the disease and improper treatment, as well as the delayed or inaccurate diagnosis and treatment, such disease brings great suffering to the patients. For the treatment of this disease, the clinical physician often prescribes the medications for anti-inflammation, analgesia, activating blood circulating and removing stasis, but the therapeutic effect is not very satisfactory.^[7–9]^ Therefore, an ideal strategy to relieve thoracic facet joint disorders is urgently needed. In recent years, traditional Chinese medicine (TCM) has been increasingly accepted by thoracic facet joint disorders patients due to its dual functions of treatment and coordinating, widely available and fewer side effects.^[10–12]^ Massage therapy, one of the most popular complementary and alternative therapies, have been used for thousands of years in China. Currently, they are increasingly used because of their lower costs and safety for clinical use.^[13,14]^

This review aims to systematically review all randomized controlled trials (RCTs) to assess the effectiveness and safety of massage treatment for patients with thoracic facet joint disorders.

## 2 Materials and methods

This systematic review protocol has been registered on OSF (DOI 10.17605/OSF.IO/XMEJD). The protocol follows the Cochrane Handbook for Systematic Reviews of Interventions and the Preferred Reporting Items for Systematic Reviews and Meta-Analysis Protocol (PRISMA-P) statement guidelines.^[15]^ We will describe the changes in our full review if needed.

## 3 Inclusion criteria for study selection

### 3.1 Type of studies

This review will include clinical RCTs of massage therapy for thoracic facet joint disorders patients without any language or publication status restrictions. Non-RCTs, quasi-RCTs, case series, case reports, crossover studies, uncontrolled trials, and laboratory studies will not be included.

### 3.2 Type of participants

Participants who were diagnosed with thoracic facet joint disorders according to related guidelines or consensus. All included participants in this review regardless of their age, race and gender. Pregnant and lactating women will be excluded.

### 3.3 Type of interventions

Interventions will include any type of clinically performed massage for improvement of thoracic facet joint disorders. This will include Chinese Massage, Japanese Massage, Thai Massage, Swedish Massage, Tuina, Shiatsu, Remedial Massage, General Massage, Acupressure, Reflexology, Manual Lymphatic Drainage. Studies of PDS combined with other interventions such as acupuncture, herbal medicines, qigong and yoga will be considered for exclusion.

Control: no intervention, treatments other than massage (e.g., usual or standard care, placebo, wait-list controls).

### 3.4 Type of outcome measures

#### 3.4.1 Main outcome(s)

The primary outcome at the end of treatment or at maximal follow-up is the clinical effective rate, which is categorized as cure, markedly effective, effective, or ineffective according to clinical symptoms, VAS score, joint mobility, daily living ability score and X-ray, etc.

#### 3.4.2 Additional outcome(s)

The secondary outcomes will include quality of life (SF-36), symptom scores (pain, limited joint mobility and joint swelling, etc), comparison of therapeutic effects under arthroscopy, and comparison of curative effect of pathological tissue, etc.

### 3.5 Search methods for the identification of studies

#### 3.5.1 Electronic searches

We will search the following electronic bibliographic databases for relevant trials:
CNKI (China National Knowledge Infrastructure Database, from 1979 to present);
Wanfang Database (from 1990 to present);
Pubmed Database (from 2000 to present);
CENTRAL (Cochrane Central Register of Controlled Trials, from 2000 to present);
CINAHL (Cumulative Index of Nursing and Allied Health Literature, from 1937 to present);
EMBASE (Excerpta Medica database, from 1947 to present);
Ovid MEDLINE ALL (Ovid Medical Literature Analysis and Retrieval System Online, from 1946 to present);

In addition, Clinical trial registries, like the Chinese Clinical Trial Registry (ChiCTR), the Netherlands National Trial Register (NTR) and ClinicalTrials.gov, will be searched for ongoing trials with unpublished data.

There will be no language restrictions.

### 3.6 Data collection and analysis

#### 3.6.1 Study identification

We will use EndNote X9 software to manage the records of searched electronic databases. The initial selection will involve scanning of the titles and abstracts of the retrieved studies. The full text of relevant studies will then be reviewed for study inclusion, in accordance with the inclusion criteria, by 2 authors (KLZ and SD). Potentially relevant articles will be reviewed independently by 2 authors to determine if they meet the prespecified criteria. Any disagreement between authors will be resolved by consensus with a third author. The study selection procedure will follow and be recorded in the PRISMA flow chart. All the evidence will be assessed by The Grading of Recommendations Assessment, Development and Evaluation (GRADE).

#### 3.6.2 Data extraction and management

According to the inclusion criteria, a standard data collection form will be made before data extraction. The following data will be extracted by 2 authors (KLZ and SD):

*General information*: Research identification, publication year, the title of the study, first author;
*Study methods*: study design, sample size, randomization method, allocation concealment, blinding, incomplete report or selecting report, other sources of bias;
*Participants*: Inclusion and exclusion criteria;
*Intervention*: motion details, treatment duration, and frequency;
*Control*: Type of control methods, motion details, treatment duration, and frequency;
*Outcomes*: Included outcome measures.

#### 3.6.3 Risk of bias assessment

The risk of bias in included studies will be assessed independently by 2 reviewers (KLZ and SD) using the Cochrane Risk of Bias Tool, with any disagreements resolved by consensus or by discussion with a third reviewer. All judgments will be fully described, and the conclusions will be presented in the Risk of Bias figures and will be incorporated into the interpretation of review findings, by means of sensitivity analysis. The risk of bias of each domain will be graded as adequate, unclear, or inadequate. We intend to use the concealment of allocation grading in investigation of any heterogeneity and in sensitivity analysis. Other aspects of study quality including the extent of blinding (if appropriate), losses to follow up, non-compliance, whether the outcome assessment was standardized, and whether an intention to treat analysis was undertaken, will be presented in the risk of bias table describing the included studies and will provide a context for discussing the reliability of the results.

#### 3.6.4 Data analysis

We will use Stata Software [Computer program] (Version 15.1) to process the meta-analysis. Weighted mean difference (WMD) will be used for continuous variable data, and the combined statistical effects of these two are combined. The X^2^ test will be adopted to analyze whether there is heterogeneity in each of the included research questions. I^2^ > 50% is a criterion for significant judgment. The fixed effect model is adopted if I^2^≤50%, which is considered to have homogeneity between the studies. The random effect model is adopted if I^2^ > 50%, which is considered to have heterogeneity among the studies. The effect size is expressed as 95% confidence interval (CI), and *P* < .05 is considered to be statistically significant.

*Sensitivity analyses:* heterogeneity may be due to the presence of 1 or more outlier studies with results that conflict with the rest of the studies. We will perform sensitivity analyses excluding outlier studies. In addition, we plan to perform sensitivity analysis to explore the influence of trial quality on effect estimates. The quality components of methodology include adequacy of generation of allocation sequence, concealment of allocation, and the use of intention-to-treat analysis.

*Meta-regression analyses:* if data permits, we will perform the meta-regression analyses.

#### 3.6.5 Publication bias

If sufficient number of trials (more than 10 trials) are found, we will generate funnel plots (effect size against standard error) to investigate publication bias.

#### 3.6.6 Ethics and dissemination

The data used in this systematic review will be collected from published studies. Based on this, the study does not require ethical approval.

## Acknowledgment

The authors thank all the researchers in our working group.

## Author contributions

**Conceptualization:** Kelin Zhou, Shuo Dong, Mingheng Cai.

**Data curation:** Shuo Dong, Mingheng Cai.

**Formal analysis:** Kelin Zhou.

**Funding acquisition:** Mingheng Cai.

**Investigation:** Wei Ji.

**Methodology:** Wei Ji, Jingyi Yang.

**Project administration:** Kelin Zhou, Sheng Guo, Mingheng Cai.

**Software:** Jingyi Yang, Mingling Ren, Baolai Mi.

**Supervision:** Sheng Guo.

**Writing – original draft:** Kelin Zhou, Shuo Dong.

**Writing – review & editing:** Sheng Guo.
